# Genome characterization and taxonomy of *Actinomyces acetigenes* sp. nov., and *Actinomyces stomatis* sp. nov., previously isolated from the human oral cavity

**DOI:** 10.1186/s12864-023-09831-2

**Published:** 2023-12-04

**Authors:** Xuechen Tian, Wee Fei Aaron Teo, Wei Yee Wee, Yixin Yang, Halah Ahmed, Nicholas S. Jakubovics, Siew Woh Choo, Geok Yuan Annie Tan

**Affiliations:** 1 Institute of Biological Sciences, Faculty of Science, Universiti Malaya, 50603 Kuala Lumpur, Malaysia; 2 Centre for Research in Biotechnology for Agriculture, Universiti Malaya, 50603 Kuala Lumpur, Malaysia; 3 School of Science, Monash University Malaysia, Jalan Lagoon Selatan, Bandar Sunway, Subang Jaya, Selangor 46150 Malaysia; 4 College of Science, Mathematics and Technology, Wenzhou-Kean University, 88 Daxue Road, Ouhai, Wenzhou, Zhejiang Province 325060 China; 5 Wenzhou Municipal Key Laboratory for Applied Biomedical and Biopharmaceutical Informatics, Wenzhou-Kean University, 88 Daxue Road, Ouhai, Wenzhou, Zhejiang Province 325060 China; 6 Zhejiang Bioinformatics International Science and Technology Cooperation Center, Wenzhou-Kean University, 88 Daxue Road, Ouhai, Wenzhou, Zhejiang Province 325060 China; 7 School of Dental Sciences, Faculty of Medical Sciences, Newcastle University, Framlington Place, Newcastle Upon Tyne, NE2 4BW UK

**Keywords:** *Actinomyces acetigenes*, *Actinomyces stomatis*, Novel species, Genome analysis, Oral cavity

## Abstract

**Background:**

*Actinomyces* strains are commonly found as part of the normal microflora on human tissue surfaces, including the oropharynx, gastrointestinal tract, and female genital tract. Understanding the diversity and characterization of *Actinomyces* species is crucial for human health, as they play an important role in dental plaque formation and biofilm-related infections. Two *Actinomyces* strains ATCC 49340^ T^ and ATCC 51655^ T^ have been utilized in various studies, but their accurate species classification and description remain unresolved.

**Results:**

To investigate the genomic properties and taxonomic status of these strains, we employed both 16S rRNA Sanger sequencing and whole-genome sequencing using the Illumina HiSeq X Ten platform with PE151 (paired-end) sequencing. Our analyses revealed that the draft genome of *Actinomyces acetigenes* ATCC 49340^ T^ was 3.27 Mbp with a 68.0% GC content, and *Actinomyces stomatis* ATCC 51655^ T^ has a genome size of 3.08 Mbp with a 68.1% GC content. Multi-locus (*atpA, rpoB, pgi, metG, gltA, gyrA*, and core genome SNPs) sequence analysis supported the phylogenetic placement of strains ATCC 51655^ T^ and ATCC 49340^ T^ as independent lineages. Digital DNA-DNA hybridization (dDDH), average nucleotide identity (ANI), and average amino acid identity (AAI) analyses indicated that both strains represented novel *Actinomyces* species, with values below the threshold for species demarcation (70% dDDH, 95% ANI and AAI). Pangenome analysis identified 5,731 gene clusters with strains ATCC 49340^ T^ and ATCC 51655^ T^ possessing 1,515 and 1,518 unique gene clusters, respectively. Additionally, genomic islands (GIs) prediction uncovered 24 putative GIs in strain ATCC 49340^ T^ and 16 in strain ATCC 51655^ T^, contributing to their genetic diversity and potential adaptive capabilities. Pathogenicity analysis highlighted the potential human pathogenicity risk associated with both strains, with several virulence-associated factors identified. CRISPR-Cas analysis exposed the presence of CRISPR and Cas genes in both strains, indicating these strains might evolve a robust defense mechanism against them.

**Conclusion:**

This study supports the classification of strains ATCC 49340^ T^ and ATCC 51655^ T^ as novel species within the *Actinomyces*, in which the name *Actinomyces acetigenes* sp. nov. (type strain ATCC 49340^ T^ = VPI D163E-3^ T^ = CCUG 34286^ T^ = CCUG 35339 ^T^) and *Actinomyces stomatis* sp. nov. (type strain ATCC 51655^ T^ = PK606^T^ = CCUG 33930^ T^) are proposed.

**Supplementary Information:**

The online version contains supplementary material available at 10.1186/s12864-023-09831-2.

## Background

Members of the genus *Actinomyces* are Gram-strain positive, anaerobic to facultatively anaerobic, rod-shaped bacteria commonly found in the human normal oral and gastrointestinal flora [1]. *Actinomyces* species are distinguished by their rod-shaped morphology and their filamentous growth pattern. They can form biofilms which play important roles in oral and dental diseases and other biofilm-related infections [2]. In the oral cavity, *Actinomyces* species are among the primary colonizers during the formation of polymicrobial biofilms such as dental plaque [3, 4]. Notable *Actinomyces* species commonly found in the oral cavity include *Actinomyces naeslundii*, *Actinomyces israelii*, *Actinomyces odontolyticus* (recently reclassified as *Schaalia odontolytica),* and *Actinomyces oris *[5]. These *Actinomyces* species, alongside other bacteria, contribute to the complex microbial communities that inhabit the oral cavity and are implicated in various oral diseases, including dental caries, periodontal diseases, and endodontic infections.

Recent advancements in phenotyping, molecular diagnostics, metagenomics, and single-cell sequencing have significantly enhanced the understanding of *Actinomyces.* These techniques have improved species identification and enabled better delineation within *Actinomyces* [5, 6]. Presently, there are thirty-three *Actinomyces* species with validly published names (https://lpsn.dsmz.de/genus/actinomyces) [7].

Strains ATCC 49340^T^ (= VPI D163E-3^T^ = CCUG 34286^ T^ = CCUG 35339^ T^) was isolated from the gingival crevice of adult with progressive periodontitis. The strain was initially identified as *Actinomyces naeslundii* serotype III [8] and later reclassified as *Actinomyces oris* following the reclassification of *Actinomyces* genospecies II [9]. The phylogenetic analyses based on multilocus sequence typing (MLST) and pilus gene sequences revealed a greater level of diversity within *Actinomyces oris* with strain CCUG 34286^ T^ forming a discrete cluster [10].

The *Actinomyces* strain PK606^T^, originally isolated from the human oral cavity, was initially identified as *Actinomyces naeslundii* [11]. Subsequently, it was preserved and made available in culture collections under the designations ATCC 51655^ T^ and CCUG 33930^ T^. The strain has been employed as a reference in numerous studies investigating the coaggregation and interactions between *Streptococcus* and *Actinomyces* species [12, 13] with studies involving the strain focused mainly on the oral biofilm sphere [14–16].

Although strains ATCC 49340^ T^ and ATCC 51655^ T^ have been utilized in various studies since 1990 [8–10, 17], their precise species classifications and descriptions remain unresolved. During our genome study on oral *Actinomyces*, we found that strains ATCC 49340^T^ (= VPI D163E-3^T^ = CCUG 34286^ T^ = CCUG 35339^ T^) and ATCC 51655^ T^ (= PK606^T^ = CCUG 33930^ T^) represent previously undescribed distinct *Actinomyces* lineages, thus we propose the names *Actinomyces acetigenes* sp. nov. and *Actinomyces stomatis* sp. nov. The description of these *Actinomyces* species would provide valuable insights into the diversity and genome characteristics of this important bacterial genus and may have practical implications for subsequent investigations of oral bacteria.

## Results

### Genome characterization of two novel species

In this study, we employed whole-genome sequencing to analyze the genomes of two strains, ATCC 49340^T^ and ATCC 51655^T^. The genome sequencing was conducted on the Illumina HiSeq X Ten platform, utilizing PE151 paired-end sequencing technology provided by the sequencing service. This advanced Illumina platform generated a substantial raw data output of 1,469 Mbp for each strain. After removing low-quality data, approximately 1,173 Mbp of clean data were obtained for each strain.

For strain ATCC 49340^T^, a total of 9,796,620 bp reads were assembled into 106 contigs (N50 = 89,254 bp, providing 358 × genome coverage) with 100% genome completeness and less than 1% contamination. The draft genome size of strain ATCC 49340^T^ is 3.27 Mb with 68.0% of GC contents. Similarly, for strain ATCC 51655^T^, a total of 9,796,512 bp reads were assembled into 63 contigs (N50 = 99,621 bp) with 381 × genome coverage, obtaining a 3.08 Mbp genome size with 68.1% of GC contents. The genome completeness is 100% and contamination is below 0.5%. The genome assembly quality for both strains exceeded 95% (Table 1), indicating high-quality assembly.

**Table 1:** Genome statistics of sequencing and assembly

**Strain Name**	**Strain ATCC 49340**^**T**^	
Raw Data (Mb)	1,469	1,469
Clean Data (Mb)	1,173	1,174
Total Reads (#)	9,796,620	9,796,512
Genome Size (Mb)	3.27	3.08
Genome Completeness (%)	100%	100%
Contamination (%)	0.95%	0.47%
Genome Quality (%)	95.25%	97.65%
Contigs (#)	106	63
N50 (bp)	89,254	99,621
GC (%)	68.0	68.1
Genome Coverage	358	381

**Fig. 1:**
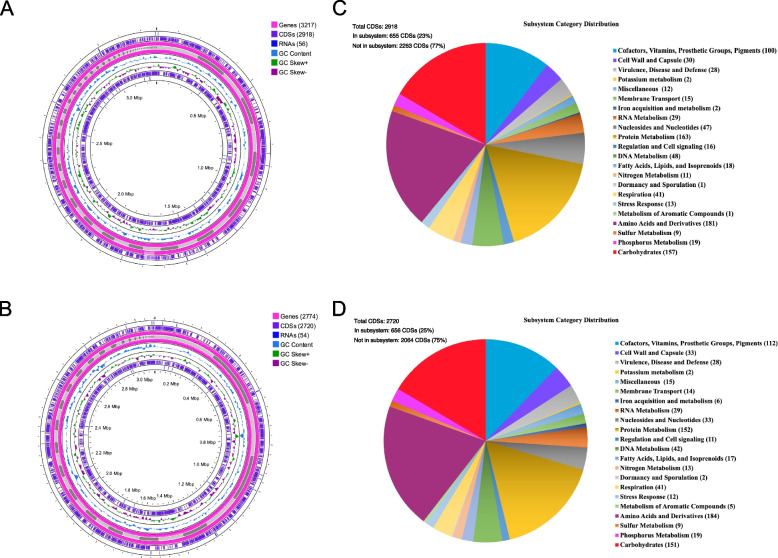
Genome annotation information. **A** A circular genomic map of strain ATCC 49340^T^, showing a circular distribution of the genes, coding sequences (CDSs), RNAs, GC content, and GC skew. **B** A circular genomic map of strain ATCC 51655^T^, showing a circular distribution of the genes, coding sequences (CDSs), RNAs, GC content, and GC skew. **C** Subsystem category distribution associated with protein-coding gene of strain ATCC 49340^T^ by RAST annotation. **D** Subsystem category distribution associated with protein-coding gene of strain ATCC 51655^T^ by RAST annotation. The circular genome maps were drawn by the Proksee online tool

The assembly genomes were annotated using the RAST web server, which identified a total of 3,217 genes, 2,918 coding sequences (CDSs), and 56 RNAs in strain ATCC 49340^T^ (Fig. 1A). For strain ATCC 51655^T^, a total of 2,774 genes, 2,720 CDSs, and 54 RNAs were identified (Fig. 1B). In strain ATCC 49340^T^, approximately 23% of CDSs matched with 224 subsystem features, which were classified into 22 categories. Similarly, strain ATCC 51655^T^ exhibited 219 annotated subsystem features, classified into the same 22 categories, accounting for 25% of CDSs. However, more than 75% of CDSs in both strains remained unassigned to any specific categories, as depicted in Figs. 1C and D.

Among these identified categories, the subsystem of amino acids and derivatives exhibited the highest number of CDSs, with 181 CDSs in strain ATCC 49340^T^ and 184 CDSs in strain ATCC 51655^T^. This was followed by the subsystem of protein metabolism, carbohydrates, and cofactors, vitamins, prosthetic groups, pigments, which accounted for 163, 157, 100 and 152, 151, 112 CDSs in strain ATCC 49340^T^ and strain ATCC 51655^T^, respectively (Fig. 1C and D). Additionally, the subsystem of virulence, disease and defense, was found in both strains with 28 CDSs. Among these, 19 CDSs were associated with resistance to antibiotics and toxic compounds, while 9 CDSs were associated with invasion and intracellular resistance.

## Phylogenetic relationships of two novel species

**Fig. 2:**
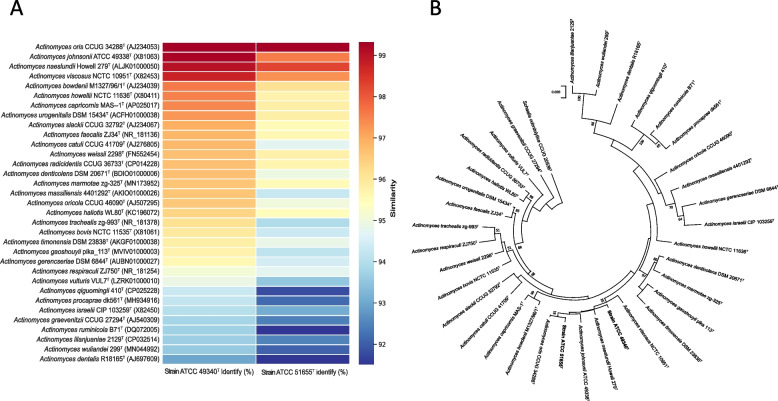
Phylogenetic analysis based on 16S rRNA gene sequences. **A** Nucleotide identity heatmap illustrating the similarity of 16S rRNA gene sequence of strains ATCC 49340^T^ and ATCC 51655^T^ compared to *Actinomyces* type strains (EzBioCloud database). Sequence similarity was calculated using the 16S-based identification tool and the pairwise nucleotide sequence alignment tool provided by EzBioCloud. **B** Maximum-likelihood tree based on 16S rRNA gene sequences of strain ATCC 49340^T^ and ATCC 51655^T^ compared to *Actinomyces* type strains (with nearly complete 16S rRNA gene sequences in NCBI database), *Schaalia odontolytica* CCUG 20536^T^ was employed as an outgroup. Bootstrap value was computed based on 1,000 bootstrap replicates, and values with more than 50% are shown. The novel species proposed in this study were highlighted in bold within the tree. Table S1 provides information on species and accession numbers used in the trees

The 16S rRNA gene sequences of strains ATCC 49340^ T^ and ATCC 51655^ T^ were validated using the Sanger sequencing method and sequences extracted from genome RAST annotation. The 16S rRNA gene sequence comparisons revealed that strain ATCC 49340^ T^ exhibited a pairwise sequence similarity of 99.4% with *Actinomyces oris* CCUG 34288^ T^, 99.3% with *Actinomyces johnsonii* ATCC 49338^ T^, 99.0% with *Actinomyces naeslundii* Howell 279^ T^, and 98.8% with *Actinomyces viscosus* NCTC 10951^ T^ (Fig. 2A). On the other hand, strain ATCC 51655^ T^ showed high pairwise sequence similarity of 99.3% with *Actinomyces oris* CCUG 34288^ T^ and 98.3% with *Actinomyces naeslundii* Howell 279^ T^ (Fig. 2A).

When examining the phylogenetic tree based on 16S rRNA gene sequence (Fig. 2B and Figure S1), strain ATCC 49340^ T^ exhibited a certainly notable genetic distance from *Actinomyces viscosus* NCTC 10951^ T^. It was also observed to be distinct from *Actinomyces oris* CCUG 34288^ T^*, Actinomyces johnsonii* ATCC 49338^ T^*, and Actinomyces naeslundii* Howell 279^ T^. Similarly, strain ATCC 51655^ T^ displayed a close relationship to *Actinomyces oris* CCUG 34288^ T^ compared to other species, yet it still exhibited some genetic divergence. These results highlighted the limitations of relying solely on phylogenetic analysis based on 16S rRNA gene sequence for precise bacterial species identification. Hence, we conducted a comprehensive genome-based analysis.

**Fig. 3:**
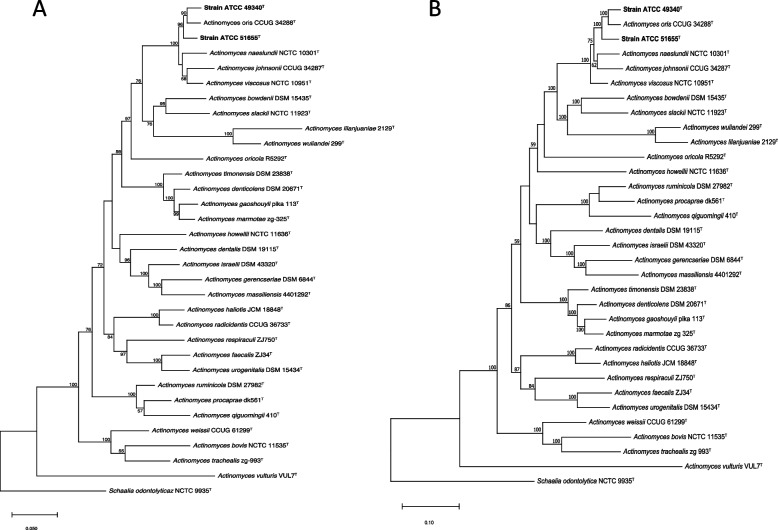
Phylogenetic analysis of strains ATCC 49340^T^ and ATCC 51655^T^ compared with *Actinomyces* type strains, based on multiple housekeeping gene and core genome SNPs sequences. **A** Phylogenomic tree based on six housekeeping gene (*atpA, rpoB, pgi, metG, gltA, and gyrA)* sequences of strains ATCC 49340^T^, ATCC 51655^T^, and other *Actinomyces* type strains. **B** Phylogenomic tree based on the concatenated nucleotide sequences of core genome SNPs of strains ATCC 49340^T^, ATCC 51655^T^, and other *Actinomyces* type strains. *Schaalia odontolytica* NCTC 9935^T^ was employed as an outgroup. The tree was constructed using the maximum-likelihood method with 1,000 bootstrap replicates, and bootstrap values above 50% are shown. The novel species proposed in this study were highlighted in bold within the tree. Table S2-3 provides all housekeeping gene and genome information (species and accession number) used in the trees

The resulting maximum-likelihood tree based on six housekeeping gene (*atpA, rpoB, pgi, metG, gltA, and gyrA*) sequences revealed a close relationship between strain ATCC 49340^T^ and *Actinomyces oris* CCUG 34288^T^, while ATCC 51655^T^ was found to be separated from the branch of *Actinomyces oris* CCUG 34288^T^ (Fig. 3A & Table S2). For all this, *Actinomyces oris* remains the closest species to strains ATCC 49340^T^ and ATCC 51655^T^. The core genome SNPs sequence analysis further supported the results obtained from the housekeeping gene analysis, indicating a consistent outcome (Fig. 3B). The collective findings from multiple genes and core genome SNPs analyses suggest that strains ATCC 49340^T^ and ATCC 51655^T^ are most closely related to *Actinomyces oris* CCUG 34288^T^. However, based on these results of the phylogenetic relationship, it is difficult to definitively conclude that strain ATCC 49340^T^ is *Actinomyces oris,* nor can we conclusively determine the identity of strain ATCC 51655^T^.

## Genome comparative analysis of two novel species

**Fig. 4:**
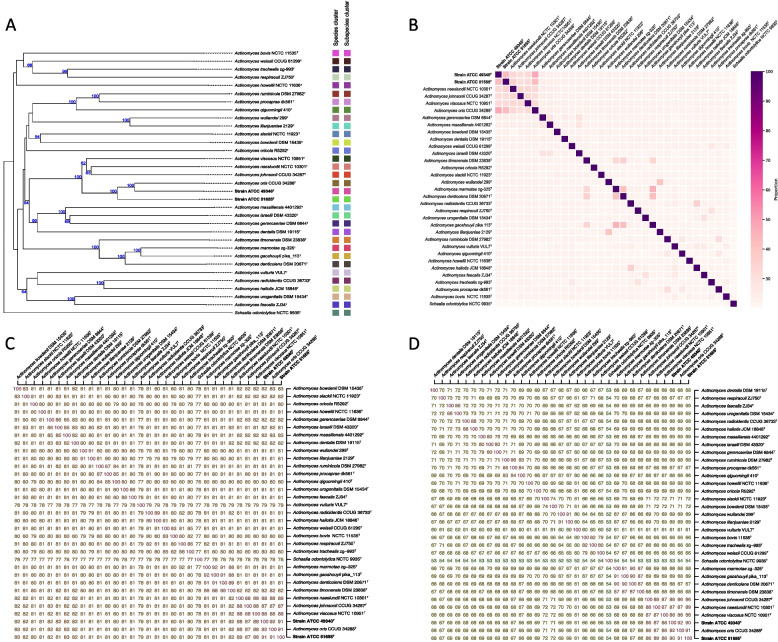
Genome comparative analysis between two sequenced strains and thirty *Actinomyces* type strains, and outgroup species of *Schaalia odontolytica* NCTC 9935^ T^. **A** Genome relationship tree based on the TYGS results, where species cluster denotes groupings formed using a 70% dDDH threshold, and subspecies cluster indicates groupings established with a more stringent 79% dDDH threshold. **B** Heatmap with dDDH value between two sequenced strains and *Actinomyces* type strains, the dDDH values were calculated based on the confidence interval of formula *d*_*4*_. **C** Matrix with ANI results between two sequenced strains and *Actinomyces* type strains, ANI values were estimated using both best hits (one-way ANI) and reciprocal best hits (two-way ANI) between two genomic datasets. **D** Matrix with AAI values between two sequenced strains and *Actinomyces* type strains, which also computed using both best hits (one-way AAI) and reciprocal best hits (two-way AAI) of each two protein datasets, all proteins sequences used in this analysis from the results of RAST annotation. The novel species proposed in this study were highlighted in bold within the tree, heatmap, and matrix

The TYGS-based analysis revealed that strain ATCC 49340^T^ is the closest to *Actinomyces oris* CCUG 34288^T^ with the highest dDDH value of 48.1% compared to other *Actinomyces* type strains (Fig. 4A, B). However, the genome tree showed a close relationship between strain ATCC 51655^T^ and *Actinomyces oris* CCUG 34288^T^, with a dDDH value of 42.4% (Fig. 4A, B). The dDDH values of both strains were below the 70% threshold for species boundary, as well as below the 79% threshold for subspecies boundary compared to the *Actinomyces* type strains (Fig. 4A), suggesting that strains ATCC 49340^T^ and ATCC 51655^T^ represent novel species.

In an expanded comparative analysis involving non-type strains of *Actinomyces oris* and *Actinomyces naeslundii* (Table S4), strains ATCC 49340^T^ and ATCC 51655^T^ were further evaluated. It was observed that non-type strains *Actinomyces oris* A19A-1, R11372, MMRCO6-1, F28B1, M48-1B-1, WE8B-23 and CCUG 34286 shared dDDH values exceeding 70% with strains ATCC 49340^T^ (Figure S2A, B), suggesting a conspecific relationship. Conversely, only *Actinomyces oris* P6N exhibited a dDDH value greater than 70% with ATCC 51655^T^, specifically 86.5%, indicating species identity. In contrast, no larger than 70% dDDH values were observed between strains ATCC 49340^T^ or ATCC 51655^T^ and any *Actinomyces naeslundii* non-type strains (Figure S2C, D), supporting the notion that these strains do not belong to the same species as any non-type strains of *Actinomyces naeslundii*. These findings affirm the potential revision of *Actinomyces oris* non-type strains classification and support the proposal of novel species status for strains ATCC 49340^T^ and ATCC 51655^T^.

To further validate the results of the digital DNA-DNA hybridization analysis results, we performed ANI and AAI analyses. As shown in Fig. 4C, D, strain ATCC 49340^T^ processed the highest ANI and AAI values of 92% against *Actinomyces oris* CCUG 34288^T^. Similarly, strain ATCC 51655^T^ exhibited ANI and AAI values of 91% against *Actinomyces oris* CCUG 34288^T^. All ANI and AAI values of strains ATCC 49340^T^ and ATCC 51655^T^ compared to all *Actinomyces* type strains, were below the specie boundary threshold of 95% to 96% [18, 19]. The genome of strains ATCC 49340^T^ and ATCC 51655^T^ were assigned to *Actinomyces* with a percentage of conserved proteins (POCP) values of 65.5% and 65.2%, respectively. A prokaryotic genus can be defined as a group of species with all pairwise POCP values higher than 50% [19]. These comparative analyses provide strong evidence that strains ATCC 49340^T^ and ATCC 51655^T^ represent novel *Actinomyces* species, warranting the proposal of new names and detailed descriptions.

**Fig. 5:**
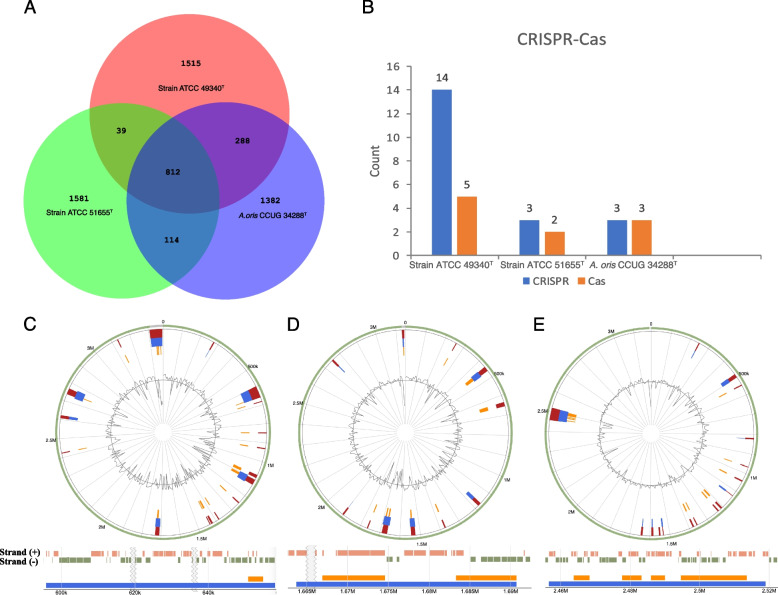
Functional prediction analysis and characterization. **A** Venn diagram showing the numbers of core genes and unique genes presented in strains ATCC 49340^T^, ATCC 51655^T^, and *Actinomyces oris* CCUG 34288^T^. **B** Visual comparison of CRISPR-Cas system in strains ATCC 49340^T^, ATCC 51655^T^, and *Actinomyces oris* CCUG 34288^T^. **C**-**E** Genomic islands (GIs) distribution and the representation of the largest GI for strains ATCC 49340^T^, ATCC 51655^T^, and *Actinomyces oris* CCUG 34288^T^, respectively. The green circle represents aligned contigs. The blue area in the circle indicates the number of GIs predicted by the IslandPath-DIMOB method. The orange area in the circle indicates the number of GIs predicted by the SIGI-HMM method, and the red area in the circle represents the gene coverage for each GI. The pink spacer indicates the gene distribution in the plus strand of the largest GI, and the green spacer represents the gene distribution in the minus strand of the largest GI

We further used *Actinomyces oris* CCUG 34288^T^ as a reference to conduct the pangenome analysis of strains ATCC 49340^T^ and ATCC 51655^T^ using Roary pangenome pipeline. The results revealed a total of 5,731 gene clusters in the pangenome of the analyzed strains, comprising 812 core genes and 4,919 shell genes (Fig. 5A). By comparing these gene clusters, we identified a total of 1,515 unique gene clusters in strain ATCC 49340^T^, distinct from strains ATCC 51655^T^ and *Actinomyces ori*s CCUG 34288^T^. Similarly, 1,518 unique gene clusters were detected in strains ATCC 51655^T^ compared to ATCC 49340^T^ and *Actinomyces ori*s CCUG 34288^T^. In contrast, *Actinomyces ori*s CCUG 34288^T^ possessed the fewest unique gene clusters with a count of 1,382. Comparison analysis of unique genes and core genes among the three strains demonstrated significant differences in the number of unique clusters between species, especially strains ATCC 49340^T^ and ATCC 51655^T^, when compared to *Actinomyces ori*s CCUG 34288^T^. This further supports their classification as novel species.

## Species functional prediction and characterization

To gain deeper insights into the properties and functions of novel species, we performed a series of analyses including genomic islands (GIs), virulence factor, pathogenicity, and CRISPR-Cas analysis by comparing to the closest type strain *Actinomyces oris* CCUG 34288^T^. The prediction of genomic islands revealed that strains ATCC 49340^T^ and ATCC 51655^T^ exhibited 24 and 16 putative GIs, respectively, while *Actinomyces oris* CCUG 34288^T^ has 22 putative GIs, and there were overlapping GIs among the three strains (Fig. 5C-E). Among the 24 GIs identified in strain ATCC 49340^T^, the largest GI was 62,295 bp, comprised of 67 genes (Fig. 5C), while the second largest was 67,552 bp, consisting of 48 genes (Table S5). For strain ATCC 51655^T^, the two largest GIs were 26,984 bp and 24,336 bp, with 24 and 20 genes, respectively (Fig. 5D and Table S5). In contrast, the top GIs of *Actinomyces oris* CCUG 34288^T^ were 62,433 bp and 19,637 bp, encompassing 64 and 24 genes, individually (Fig. 5E and Table S5). GIs are the gene cluster associated with the horizontal origin of the prokaryotic genome and the adaptive to the environment [20]. These differences in GIs between strains ATCC 49340^T^ and ATCC 51655^T^ compared to *Actinomyces oris* CCUG 34288^T^ are obvious, providing further evidence for classification as novel species.

The pathogenic analysis (Table S6) revealed that strains ATCC 49340^T^ and ATCC 51655^T^, as well as *Actinomyces oris* CCUG 34288^T^, exhibit genomic features that align with those found in the pathogenic family which includes *Cutibacterium acnes* (formerly *Propionibacterium acnes),* within the class *Actinomycetes*, indicating that their human pathogenicity risks.

To further assess their pathogenic potential, we conducted a prediction of their potential virulence factors using the Virulence Factor Database (VFDB). This database is a valuable resource for identifying genes associated with virulence in bacteria. By comparing the genomes of the three *Actinomyces strains* (ATCC 49340^T^, ATCC 51655^T^, and CCUG 34288^T^) against 32,672 virulence factors in the VFDB database, we found that 29 virulence-associated factors in strain ATCC 49340^ T^, these factors mainly including stress survival (*pafA, mpa, ahpC*), Immune modulation (*nuoG, rmlA*), Regulation (*phoR, sigA/rpoV, sigH*), Adherence (*ABG47036, srtC1, fimP, fimQ, groEL2, AvisC_010100012015, AvisC_010100012020*), Nutritional/Metabolic factor (*lysA, pvdL, ctpV, narX, phzC1, narG, phzC2, sugC, narH, leuD, glnA1*), Effector delivery system (*tssH/clpV1*), and Exoenzyme (*zmp1*). Similarly, in strain ATCC 51655^ T^, a total of 28 genes encoding virulence-associated factors were identified*,* including stress survival* (mpa, ahpC, pafA),* Immune modulation* (nuoG, rmlA*), Regulation (*phoR, sigA/rpoV, sigH, relA, regX3*), Adherence (*ABG47036, fimP, AvisC_010100012025, srtC1, fimQ, groEL2, AvisC_010100012020*), Nutritional/Metabolic factor (*DDA3937_RS14675, VF0849, ctpV, sugC, narG, VF0302, narH, glnA1, ctpC*), Effector delivery system (*tssH/clpV1*), and Exoenzyme (*zmp1*).

In contrast, *Actinomyces oris* CCUG 34288^ T^ harbors 31 genes encoding virulence-associated factors, including 3 genes associated with stress survival *(mpa, ahpC, pafA),* 2 genes matched to Immune modulation* (nuoG, rmlA),* 4 genes matched to Regulation (*phoR, sigA/rpoV, sigH, regX3*), 2 genes assigned to Exotoxin (*cyaB, rtxB*), 1 gene assigned to Effector delivery system (*xcpR*), and 8 genes matched to Adherence, and 11 genes assigned to Nutritional/Metabolic factor (Table S9). Notably, the absence of exotoxin-associated genes (*cyaB* and *rtxB*) in strains ATCC 49340^T^ and ATCC 51655^T^ compared to strain *Actinomyces oris* CCUG 34288^ T^ suggests that two novel strains may lack the ability to produce extracellular exotoxin, potentially influencing their infection mechanisms and pathogenicity. However, the presence of the exoenzyme-associated gene *zmp1*(encoding putative zinc-dependent metalloprotease-1) in two novel strains, but not in stain *Actinomyces oris* CCUG 34288^ T^ implies variations in their interaction with the host, which could impact their infection mechanisms and pathogenicity, and may lead to different pathological processes and clinical manifestations. The complete list of virulence factors can be found in Table S9.

Further CRISPR-Cas analysis exposed that 14 CRISPRs and 5 Cas genes were identified in strain ATCC 49340^ T^, with the largest CRISPR array consisting of 87 spacers. In contrast, stain ATCC 51655^ T^ exhibited only 3 CRISPR and 2 Cas genes, and the largest CRISPR array consisted of only 2 spacers (Fig. 5B and Table S7). *Actinomyces oris* CCUG 34288^ T^ possessed 3 CRISPR and 3 Cas genes. The number of CRISPR arrays in strain ATCC 49340^ T^ was approximately five times higher than that in strain ATCC 51655^ T^ and *Actinomyces oris* CCUG 34288^ T^, and it also hosted a greater number of Cas type and subtypes (Fig. 5B and Table S8). Notably, only one Class 2 CRISPR-Cas system was identified in strain ATCC 49340^ T^, and none were identified in the other two analyzed strains, suggesting that strain ATCC 49340^ T^ may have significant functional defenses compared to the other two strains. The CRISPR-Cas system serves as an adaptive immune system found in bacteria, providing protection against foreign genetic elements such as bacteriophages and plasmids. CRISPR-Cas analysis suggests that strain ATCC 49340^ T^ might have encountered a wide variety of genetic invaders in its environment and evolved a robust defense mechanism against them, as indicated by its higher number of CRISPR-Cas genes compared to strain ATCC 51655^ T^ and *Actinomyces oris* CCUG 34288^ T^. A higher number of CRISPR-Cas genes signify a greater potential for adaptability and survival in challenging environments.

## Discussion

The whole-genome sequencing approach allowed us to obtain high-quality draft genomes for strains ATCC 49340^ T^ and ATCC 51655^ T^, with a genome completeness of 100% and low contamination levels. The genome size of ATCC 49340^ T^ is 3.27 Mb, while ATCC 51655^ T^ has a genome size of 3.08 Mb, with similar GC contents in two strains of approximately 68%. Annotation of the genomes provided insights into the coding sequences (CDSs) and various subsystem features.

Phylogenetic analysis based on the 16S rRNA gene sequence confirmed the close relationship between strain ATCC 49340^ T^ and *Actinomyces oris* CCUG 34288^ T^, while strain ATCC 51655^ T^ showed a close identity with *Actinomyces oris* CCUG 34288^ T^ but exhibited some genetic divergence. Although strain ATCC 49340^ T^ exhibits a close relationship with *Actinomyces oris* CCUG 34288^ T^ based on the 16S rRNA gene sequence, it also shows high similarity (> 99%) to *Actinomyces naeslundii* Howell 279^ T^ and *Actinomyces johnsonii* ATCC 49338^ T^. This highlights the challenge of accurately identifying the species taxonomy of strain ATCC 49340^ T^. Therefore, relying solely on 16S rRNA gene sequence analysis for species identification has limitations [10]. To overcome these limitations, we employed multiple gene analysis and core genome SNPs analysis, which consistently supported the classification of strains ATCC 49340^ T^ and ATCC 51655^ T^ as novel *Actinomyces* species, with *Actinomyces oris* CCUG 34288^ T^ being the closest species. However, strain ATCC 49340^ T^ did not separate from the branch of *Actinomyces oris* CCUG 34288^ T^. Thus it is important to acknowledge that housekeeping genes and core genome SNPs may not readily differentiate all genera or new strains of *Actinomyces*, and different studies may yield varying results using these approaches. Although core genome SNPs is a good marker to identify genome polymorphisms, which are associated with the species' phylogenetic relation [21]. In our case, the results obtained from the housekeeping gene and core genome SNPs analysis were consistent, indicating that the two approaches may be stable for the identity identification of some species.

Through employing various genomic analysis approaches, including the Type Strain Genome Server (TYGS), Average Nucleotide Identity (ANI), Average Amino Acid Identity (AAI), and pangenome analysis, we gained valuable insights into the genetic characteristics and evolutionary relationships of two novel *Actinomyces* strains. The TYGS analysis revealed that the dDDH values for strains ATCC 49340^ T^ and ATCC 51655^ T^ fell below the threshold for species boundary (70%) compared to all available genomes of *Actinomyces* type strains, suggesting that strains ATCC 49340^ T^ and ATCC 51655^ T^ represent novel *Actinomyces* species. Further comparison with non-type strains of *Actinomyces oris* and *Actinomyces naeslundii* revealed that non-type strains such as *Actinomyces oris* A19A-1, R11372, MMRCO6-1, F28B1, M48-1B-1, WE8B-23, and CCUG 34286 are conspecific with strain ATCC 49340^ T^, and *Actinomyces oris* P6N is conspecific with strain ATCC 51655^ T^. Mughal et al. (2023) reported several subgroups within *Actinomyces oris*, where strains P6N, OT171, and CCUG 33920 were grouped into a primary cluster, while stains CCUG 34286, A19A-1, and R11372 were categorized into *A. oris* 1, and strains F28B1, M48-1B-1, and WE8B-23 into *A. oris* 2 [17]. Unfortunately, these strains have not yet been effectively classified and named with representative-type strains. Based on our findings, we propose that strain ATCC 49340^ T^ should be designated as the type strain of the new species, clarifying the taxonomic status of A19A-1, R11372, MMRCO6-1, F28B1, M48-1B-1, WE8B-23, and CCUG 34286. Similarly, ATCC 51655^ T^ should represent the type strain for *Actinomyces oris* P6N, affirming its taxonomic identity.

The ANI and AAI values obtained in our study were consistent with the TYGS analysis and provided additional evidence supporting the novel species status of both strains. The dDDH and ANI approach has been widely accepted as the gold standard for differentiating species within the same genus [22–24]. Although the boundary value of dDDH and ANI remains somewhat controversial, a dDDH value below 70% corresponds to an ANI value below 95–96% have been referred for more than ten years, indicating their reliability in novel species identification [22]. Species classification was additionally confirmed through digital DNA-DNA hybridization, ANI, and AAI approaches, further highlighting that 16S sequencing methods, multilocus sequence typing (MLST) such as housekeeping gene and core genome SNPs are insufficient in discriminating the species identity [9, 10, 17].

Furthermore, comparative genome analysis revealed a pangenome consisting of 5,731 gene clusters, including 812 core gene clusters and 4,919 shell gene clusters. The identification of unique gene clusters in strains ATCC 49340^ T^ and ATCC 51655^ T^, compared to *Actinomyces oris* CCUG 34288^ T^, provided additional support for their classification as novel species. These unique genes likely contribute to the phenotype properties and functional diversity of a particular strain. These genes may encode proteins or enzymes involved in specialized metabolic pathways, stress responses, or virulence factors that enable the strain to adapt to specific ecological niches or interact with the host organism [25].

Additionally, there were differences in the presence of genomic islands between the two novel strains and *Actinomyces oris* CCUG 34288^ T^, providing further evidence for their distinct species status. Genomic islands contribute to genetic diversity within a species or strain by introducing novel genetic material from other organisms, enhancing the adaptability and evolutionary potential of the host organism [26]. Meanwhile, genomic islands often contain genes that confer specific adaptive traits, such as the ability to utilize new nutrient sources or survive in particular environments. Their acquisition can improve the fitness and competitiveness of the host organism under specific conditions [27]. A larger number of genomic islands may indicate a higher degree of genomic plasticity, increased potential for horizontal gene transfer, and potentially greater adaptive capabilities, enhancing the organism's ability to respond to changes in the environment or to colonize different niches [28]. Genomic islands play a significant role in shaping the genetic makeup and adaptive potential of organisms. The presence, content, and number of genomic islands can influence the organism's fitness, pathogenicity, antibiotic resistance, and overall ability to adapt to different environments [26].

Pathogenicity analysis indicated that strains ATCC 49340^ T^, ATCC 51655^ T^, and *Actinomyces oris* CCUG 34288^ T^ have genomic features that are commonly associated with pathogenic bacteria. These strains showed genomic similarities to organisms in the same genomic clade as *Cutibacterium acnes* (formerly *Propionibacterium acnes*), which is known for its role in human skin conditions such as acne. These findings suggest that strains ATCC 49340^ T^ and ATCC 51655^ T^ may pose a potential risk as a pathogen. Further prediction of virulence factors revealed specific genes associated with stress survival, immune modulation, regulation, adherence, nutritional/metabolic factors, and effector delivery systems, in strains ATCC 49340^ T^, ATCC 51655^ T^, and *Actinomyces oris* CCUG 34288^ T^. The absence of exotoxin-associated genes (*cyaB* and *rtxB*) in strains ATCC 49340^T^ and ATCC 51655^T^ suggests that two novel strains may lack the ability to produce extracellular exotoxin, potentially influencing their infection mechanisms and pathogenicity.

The CRISPR-Cas system serves as an adaptive immune system found in bacteria, providing protection against foreign genetic elements such as bacteriophages and plasmids. It accomplishes this by capturing and integrating fragments of foreign DNA into the bacterial genome as spacer sequences. These spacer sequences are transcribed into small RNA molecules, which guide the Cas proteins to recognize and degrade the corresponding foreign DNA during subsequent infection [29–31]. Our CRISPR-Cas analysis showed that strain ATCC 49340^ T^ has a higher number of CRISPR-Cas genes compared to strain ATCC 51655^ T^ and *Actinomyces oris* CCUG 34288^ T^. This suggests that strain ATCC 49340^ T^ may have encountered a wide variety of genetic invaders in its environment and has evolved a robust defense mechanism against them. The higher number of CRISPR-Cas genes signifies a greater potential for adaptability and survival in challenging environments.

However, It is important to acknowledge that the limitations of this study might include (i) The genomes were not fully assembled into single contigs. Even though the coverage was deemed 100% complete for each strain, there is a possibility that some genetic information was not included in the assemblies; (ii) Virulence factor analysis is context-dependent. Genes involved in functions such as stress survival, metabolism and adhesion would be important to promote infection, but they could also be beneficial if they are expressed by a commensal strain. Therefore, bioinformatics analysis alone cannot easily predict pathogenic potential; (iii) Each of the novel species is represented by only one genome sequence, additional sequencing will provide more detailed insights into these species.

In recent years, genome-based taxonomic classification and prokaryote description-based-sequence data have been widely used for species classification and the description of novel descriptions [32, 33]. Comparative genomics provides a comprehensive approach to reveal differences among bacterial strains at multiple aspects, including genome size, GC content, dDDH value, ANI value, AAI value, genomic islands, core and unique genes, virulence factors, CRISPR-Cas systems, and more. This method is considered to be more accurate and precise in identifying and classifying novel species compared to traditional phenotype observations, 16S rRNA gene analysis, and multi-locus sequence typing methods. By examining various genomic features and characteristics, comparative genomics enables a deeper understanding of the genetic diversity and evolutionary relationships among different strains, ultimately leading to more accurate species identification and classification.

## Conclusion

Whole-genome sequencing and comparative genomics analyses supported the classification of strains ATCC 49340^ T^ and ATCC 51655^ T^ as novel species, with *Actinomyces oris* CCUG 34288^ T^ being the closest species. The presence of unique genes and differences in genomic islands further validated their status as novel species. Pathogenicity analysis reveals their pathogenic nature and their association with human infection. Further CRISPR-Cas analysis revealed their adaptive immune system and their potential roles in defending against foreign genetic elements. In summary, *Actinomyces acetigenes* sp. nov. *and Actinomyces stomatis* sp. nov., are proposed with a detailed description as below, contributing to our understanding of *Actinomyces* diversity and highlighting the importance of genomic analysis in identifying and characterizing novel bacterial species. Further investigations into the functional roles of the identified unique genes and virulence factors will provide valuable insights into the pathogenic potential of these novel *Actinomyces* species.

## Description of *Actinomyces acetigenes* sp. nov.

*Actinomyces acetigenes* (a.ce.ti.ge’nes. L. neut. n. *acetum*, vinegar; Gr. ind. v. *gennaô*, to produce; N.L. part. adj. *acetigenes*, producing acetate).

The characteristics are as given by Johnson et al. [8] and Henssge et al. [9]. Additional genomic characterisations are, utilise arbutin, salicin and starch as carbon sources, the presence of pathways for acetate (EC:2.3.1.8, 2.7.2.1) and propionate (EC:2.3.1.8, 2.7.2.1) productions, utilise sulfide and L-serine to produce L-cysteine and acetate (EC:2.3.1.30, 2.5.1.47), presence of sulfate assimilatory reduction pathway (EC:2.7.7.4, 2.7.1.25, 1.8.4.8, 1.8.7.1), and produce riboflavin (vitamin B2) (EC:3.5.4.25, 3.5.4.26, 1.1.1.193, 3.1.3.104, 4.1.99.12, 2.5.1.78, 2.5.1.9, 2.7.1.26, 2.7.7.2).

The type strain ATCC 49340^T^ (= VPI D163E-3^T^ = CCUG 34286^T^ = CCUG 35339^ T^) has a genome size of 3.27 Mbp with a GC content of 68.0%, Strain VPI D163E-3^T^ was isolated from gingival crevice of adult with progressive periodontitis.

## Description of *Actinomyces stomatis* sp. nov.

*Actinomyces stomatis (*sto.ma’tis. Gr. neut. n. *stoma* (gen. *stomatos*), mouth; N.L. gen. neut. n. *stomatis*, of the mouth).

The characteristics are as given by Kolenbrander [12, 13, 34] and BacDive (https://bacdive.dsmz.de/strain/147639) [35]. Additional genomic characterizations are, utilise arbutin, salicin and starch as carbon sources, presence of pathways for acetate (EC:2.3.1.8, 2.7.2.1) and propionate (EC:2.3.1.8, 2.7.2.1) productions, utilises sulfide and L-serine to produce L-cysteine and acetate (EC:2.3.1.30, 2.5.1.47), and presence of sulfate assimilatory reduction pathway (EC:2.7.7.4, 2.7.1.25, 1.8.4.8, 1.8.7.1).

The type strain, ATCC 51655^T^ (= PK606^T^ = CCUG 33930^T^) has a genome size of 3.08Mbp with a GC content of 68.1%. Strain PK606^T^ was isolated from the human oral cavity.

## Materials and methods

### Sample collection and preparation

The strains ATCC 49340^T^ and ATCC 51655^T^ utilized in this study were originally sourced from the American Type Culture Collection (ATCC) and are currently maintained in Dr. Nicholas S. Jakbovics’ laboratory at Newcastle University, United Kingdom. The strains were routinely maintained in BHYE broth (37 g/L of brain heart infusion + 5 g/L of yeast extract, pH = 7.5) with an anaerobic growth condition, and were long-term stored at -80 °C with 40% glycerol.

## Genomic DNA extraction

Bacterial cells were harvested from BHYE broth cultures grown for 18 h at 37 °C and then re-suspended in 150 μl (37 °C pre-warmed) spheroplasting buffer (20 mM Tris–HCl, pH 6.8; 10 mM MgCl_2_; 26% w/v raffinose.5H_2_O) containing 250 μg/mL lysozyme (Sigma Aldrich) and 5 μg mutanolysin (Sigma Aldrich, reconstituted at 10 U mL^−1^). Cells were incubated at 37 °C for 30 min. Cells were transferred to screw-cap tubes containing 150 μl of 2 × T&C Lysis Solution from the MasterPure™ Gram Positive DNA Purification Kit (Epicentre®, Cat. No. MCD85201) and 15-50 mg of acid-washed glass beads (0.1 mm). Cells were lysed by bead beating in a Qiagen Tissue Lyser (Qiagen, Manchester, UK) at 50 Hz, for 5 min. Genomic DNA was then purified with the MasterPure™ Gram Positive DNA Purification Kit following the manufacturer’s instructions. In the final step, DNA was suspended in 25 μl elution buffer (10 mM Tris pH 8.5). The concentration of DNA was determined using a NanoDrop Spectrophotometer (Thermo Scientific), DNA integrity and purity were detected using agarose gel electrophoresis with a 1% agarose gel at 100 V for 40 min.

## Gene amplification and Sanger sequencing

To amplify the 16S rRNA gene sequence, we employed a previously described method [36]. PCR amplification and Sanger sequencing were conducted using universal primers 27F (5'-AGAGTTTGATCMTGGCTCAG-3') and 1492R (5 ‘-TACGGYTACCTTGTTACGACTT-3'). The PCR products were purified and amplicon sequencing was carried out by 1st Base Company (Malaysia). The 16S rRNA gene sequences were then analyzed using the EzBioCloud web server [37].

## Library construction and genome sequencing

High-quality genomic DNA was utilized for library construction. For fragmentation, 1 μg of genomic DNA was randomly fragmented using Covaris S2 for 120 s at 5.5–6.0 °C. The resulting fragmented DNA was then selected for an average size of 200–400 base pairs using magnetic beads and quantified using a Qubit fluorometer (Thermo Fisher Scientific). Next, the DNA fragments underwent end-repair and 3' adenylation using T4 DNA polymerase, Klenow DNA polymerase, and T4 polynucleotide kinase. Adapter sequences were subsequently ligated to the blunt ends of the 3' adenylated fragments. The ligated products containing the adapter sequences were selected using 2% agarose gels (Invitrogen) and amplified through polymerase chain reaction (PCR). Following purification of the PCR products, the double-stranded PCR products were heat denatured and circularized using the split oligo sequence. The library was then prepared using the single-strand circular DNA and assessed for quality using an Agilent BioAnalyzer 2100. Subsequently, the final library was subjected to sequencing on an Illumina HiSeq X Ten platform using PE151 (paired-end) sequencing for 150 bp, following the manufacturer's recommended protocols (Illumina). The resulting raw sequence data were generated in fastq format, allowing for subsequent analysis and application in the study.

## Data processing, genome assembly and annotation

To obtain high-quality sequences for subsequent analysis, the raw sequencing data were filtered and removed lower quality data (e.g. Phred quality score of < 20, reads with a ≥ 10% of Ns, adapter and duplication contamination) using a standalone PRINSEQ lite [38]. Clean reads were generated in FASTA format for genome assembly. A flexible bacteria genome analysis pipeline (Bactopia v 1.7.1) was used to assemble the sequenced data [39]. The assembled genome sequences were evaluated using the Quality Assessment Tool for Genome Assemblies (QUAST: https://quast.sourceforge.net) [40], and the genome completeness assessment was conducted through CheckM (v 1.2.2) tool [41]. To further evaluate and verify the genome assembly quality, gVolante2 [42, 43], a more accurate genome assembly assessment online interface, was used based on BUSCO v5 tool [44–46]. All assembled genomes with high quality were annotated using the RAST webserver (https://rast.nmpdr.org), a rapid annotation search tool for bacteria genes annotation [47–49]. For consistency, thirty available reference genomes of the *Actinomyces* type strain (with valid published and correct names) were also downloaded from the National Center of Biotechnology Information (NCBI) RefSeq [50] database (https://www.ncbi.nlm.nih.gov) to perform the same annotation using RAST.

## Phylogenetic analyses

To infer the *Actinomyces* phylogeny, multiple approaches were employed according to previously described methods [20]. Briefly, the 16S rRNA gene sequences of two sequenced strains were obtained and confirmed using the 16S Sanger sequencing approach and RAST annotation with genome sequences. To determine the preliminary identities of the strains, the 16S rRNA gene sequences were analyzed using the 16S-based identification tool and the pairwise nucleotide sequence alignment tool provided by EzBioCloud [37]. The 16S rRNA gene sequence of thirty-three *Actinomyces* type strains, as well as *Schaalia odontolytica* CCUG 20536^ T^ were also downloaded from the NCBI database, to construct the 16S phylogenetic tree. Similarly, six housekeeping genes (*atpA*: ATP synthase subunit alpha*, rpoB*: RNA polymerase, β-subunit*, pgi*: glucose-6-phosphate isomerase*, metG*: methionyl-tRNA synthetase*, gltA*: citrate synthase I*, and gyrA*: DNA gyrase subunit A*)* extracted from genome annotation and concatenated into a long sequence were employed for multi-locus sequence analysis [9]. For the core genome single-nucleotide polymorphisms (SNPs) analysis, the Pan-genome Sequence Analysis (PanSeq) pipeline was used for the extraction and identification of the core genome regions and SNPs from each genome sequence [51]. Default parameters were used, except the percentage identity cutoff of the core genome SNPs was 50% and the core genome threshold was confirmed as 33 (the number of genomes employed including outgroup). The core genome SNP sequences of all genomes used were merged into a file for building a more robust phylogenetic tree. The MEGA X (v 10.2.6) software was used to align all sequences based on the Muscle algorithm with default settings. The maximum likelihood (ML) tree inferred was based on Kimura two-parameter model with 1,000 bootstrap replicates, and two more trees were built based on the neighbor-joining and maximum parsimony methods using MEGA with 1000 bootstraps [52, 53]. In addition, the type strain genome server (TYGS) was employed to perform the genome comparison between two sequenced strains and thirty available *Actinomyces* type strain genomes, as well as the outgroup genome of *Schaalia odontolytica* CCUG 20536^ T^.

## Overall genome relatedness index

The overall genome relatedness index (OGRI) was employed to evaluate the genome of strains ATCC 49340^T^ and ATCC 51655^T^ with available *Actinomyces* type and non-type strain genomes using the average nucleotide identify (ANI), average amino acid identity (AAI) and digital DNA-DNA hybridization (DDH). The digital DNA-DNA hybridization (dDDH) value was calculated using TYGS webserver [54] and with a genome-based tree inferred with FastME 2.1.6.1 from GBDP distances calculated from genome sequences [55]. A heatmap with the dDDH results was built by the GSP 6.0 Heatmap Illustrator (Heml 2.0) tool [56]. The threshold of the dDDH value was set as less than 70% for species identity and less than 79% for subspecies identity according to previous research [18, 57, 58]. The ANI and AAI analyses were performed with the all-vs-all distances in all uploaded genome and protein sequences based on both one-way ANI or AAI (best hits) and two-way ANI or AAI (reciprocal best hits) using the ANI/AAI Matrix online tool developed by Kostas lab with the recommended parameter settings [57, 59]. The species delineation threshold was 95 to 96% ANI and AAI [18, 20, 60].

## Pangenome analysis

To describe the complete gene set of the most closely related species, including the core genome and unique genome, the pangenome pipeline (Galaxy Version 3.13.0 + galaxy2) was employed to conduct a pangenome analysis [61]. The genome files were uploaded to Prokka Prokaryotic genome annotation (Galaxy Version 1.14.6 + galaxy1) with default job resource parameters to generate the gff3 files [62, 63]. The gff3 files of strains ATCC 49340^T^ and ATCC 51655^T^, as well as *Actinomyces oris* CCUG 34288^ T^ were then input into the Roary pipeline. The minimum percentage identity for blastp and the percentage of isolates a gene must be in to be core were set at 95% and 99%, respectively, with the other job resource parameters using default settings. The comparison results were visualized using BioVenn online tool [64].

## Genomic islands (GIs) prediction

To perform genomic islands (GIs) analysis, the closest species to two study strains according to the genome tree, *Actinomyces oris* CCUG 34288^ T^ was used for comparison. The Genebank files generated via RAST annotation were utilized for the prediction of genomic islands (GIs) using the IslandViewer web server (https://www.pathogenomics.sfu.ca/islandviewer/upload/) as described previously [65]. *Actinomyces oris* strain T14V was selected as the reference genome to align the sequenced genome against. Genome contigs were reordered by aligning them with the reference genome using the Mauve tool [66]. The reordered sequences and genomic locations of GIs were then downloaded for interpretation purposes.

## Virulence factor prediction, pathogen analysis, and CRISPR-Cas analysis

Type strain genome of the closest species *Actinomyces oris* CCUG 34288^ T^ and two sequenced genomes in this study were analysis for the presence of virulence factors by the Virulence Factor Database (VFDB) [67, 68]. In brief, the nucleotide sequences of three genomes were searched against 32,672 nucleotide sequences (accessed on 20 June 2023) from VFDB full dataset (setB) with default parameters. Hits with an identity of more than 70% were selected in the results [69]. PathogenFinder 1.1 online tool [70] was employed to conduct a bacterial pathogenicity estimation. The assembled fasta files were uploaded to PathogenFinder, and *Actinobacteria* was selected as the organism class. For CRISPR-Cas analysis, the genome sequences of strain ATCC 49340^ T^, ATCC 51655^ T^, and *Actinomyces oris* CCUG 34288^ T^ were uploaded to CRISPR-Cas +  + online tool [71]. The CRISPRs and Cas genes were detected using the CRISPRCasMeta program (https://crisprcas.i2bc.paris-saclay.fr/CrisprCasMeta/Index).

## Protologue for descriptions of the new species

Additional taxonomic, functional and ecological features used to describe the novel species were based on the output of Protologger (http://www.protologger.de/) [72]. The formal description (protologue) was included in compliance with the requirement of the International Code of Nomenclature of Prokaryotes (2022 Revision) Rule 27 [73].

### Supplementary Information

**Additional file 1.** 

## Data Availability

The genome sequence and 16S rRNA gene sequence of strain ATCC 49340 ^T^ and ATCC 51655 ^T^ were submitted to the GenBank database. The 16S rRNA gene sequences for strain ATCC 49340 ^T^ and ATCC 51655 ^T^ were assigned accession numbers OQ981482 and OQ981481 respectively. The accession number of whole genome sequences for strain ATCC 49340 ^T^ and ATCC 51655 ^T^ were assigned as JASPFC000000000 and JASPEP000000000, respectively. These genome sequences can be accessed by searching PRJNA976213 in the NCBI database ( https://www.ncbi.nlm.nih.gov/). The accession number of other 16S rRNA gene sequences and genome sequences used in this study can be found in Table S1 and Table S3, respectively. Other any additional data or information please refer to the author: https://www.tianxuechen@wku.edu.cn.
