# Supplementary material for: Genome characterization and taxonomy of Actinomyces acetigenes sp. nov., and Actinomyces stomatis sp. nov., previously isolated from the human oral cavity
Source: BMC Genomics. 2023 Dec 4;24:734. doi: 10.1186/s12864-023-09831-2 (PMC10696680; doi:10.1186/s12864-023-09831-2)
Supplement: Supplementary file 1 — Additional file 1. [file 12864_2023_9831_MOESM1_ESM.zip › Supplementary Information.docx]

**Supplementary Information**

Xuechen Tian^1^, Wee Fei Aaron Teo^1,2^, Wei Yee Wee^3^, Yixin Yang^4,5,6^, Halah Ahmed^7^, Nicholas S. Jakubovics^7*^, Siew Woh Choo^4,5,^^6*^ and Geok Yuan Annie Tan^1,2*^

^1^Institute of Biological Sciences, Faculty of Science, Universiti Malaya, 50603 Kuala Lumpur, Malaysia.

^2^Centre for Research in Biotechnology for Agriculture, Universiti Malaya, 50603 Kuala Lumpur, Malaysia.

^3^School of Science, Monash University Malaysia, 46150 Bandar Sunway, Malaysia.

^4^College of Science, Mathematics and Technology, Wenzhou-Kean University, 88 Daxue Road, Ouhai, Wenzhou, Zhejiang Province, 325060 China.

^5^Wenzhou Municipal Key Laboratory for Applied Biomedical and Biopharmaceutical Informatics, Wenzhou-Kean University, Ouhai, Wenzhou, Zhejiang Province, 325060 China.

^6^Zhejiang Bioinformatics International Science and Technology Cooperation Center, Wenzhou-Kean University, Ouhai, Wenzhou, Zhejiang Province, 325060 China.

^7^School of Dental Sciences, Faculty of Medical Sciences, Newcastle University, Framlington Place, Newcastle upon Tyne, NE2 4BW, UK.

*Corresponding authors:

Geok Yuan Annie Tan; Email: [gyatan@um.edu.my](mailto:gyatan@um.edu.my); Institute of Biological Sciences, Faculty of Science, University of Malaya, 50603 Kuala Lumpur, Malaysia

Nicholas S. Jakubovics; Email: [nick.jakubovics@newcastle.ac.uk](mailto:nick.jakubovics@newcastle.ac.uk); School of Dental Sciences, Faculty of Medical Sciences, Newcastle University, Framlington Place, Newcastle upon Tyne, NE2 4BW, UK

Siew Woh Choo; Email: [cwoh@wku.edu.cn](mailto:cwoh@wku.edu.cn); College of Science, Mathematics and Technology, Wenzhou-Kean University, 88 Daxue Road, Ouhai, Wenzhou, Zhejiang Province, 325060 China

**Figure S1.Phylogenetic tree based on 16S rRNA gene sequences.** (A) A neighbor-joining phylogenetic tree based on 16S rRNA gene sequences of strains ATCC 49340^T^ and ATCC 51655^T^ compared to the type strains of *Actinomyces* species, the tree was inferred using the neighbor-joining method with Kimura 2-parameter model. (B) A maximum parsimony phylogenetic tree based on 16S rRNA gene sequences of strains ATCC 49340^T^ and ATCC 51655^T^ compared to type strains of *Actinomyces* species, the tree was inferred using the maximum parsimony method with subtree-pruning-regrafting (SPR) model. *Schaalia odontolytica* CCUG 20536^T^ was employed as an outgroup in two trees. Bootstrap value was computed based on 1000 bootstrap replicates, and values with more than 50% are shown. The novel species proposed in this study were highlighted in bold within the tree.

**
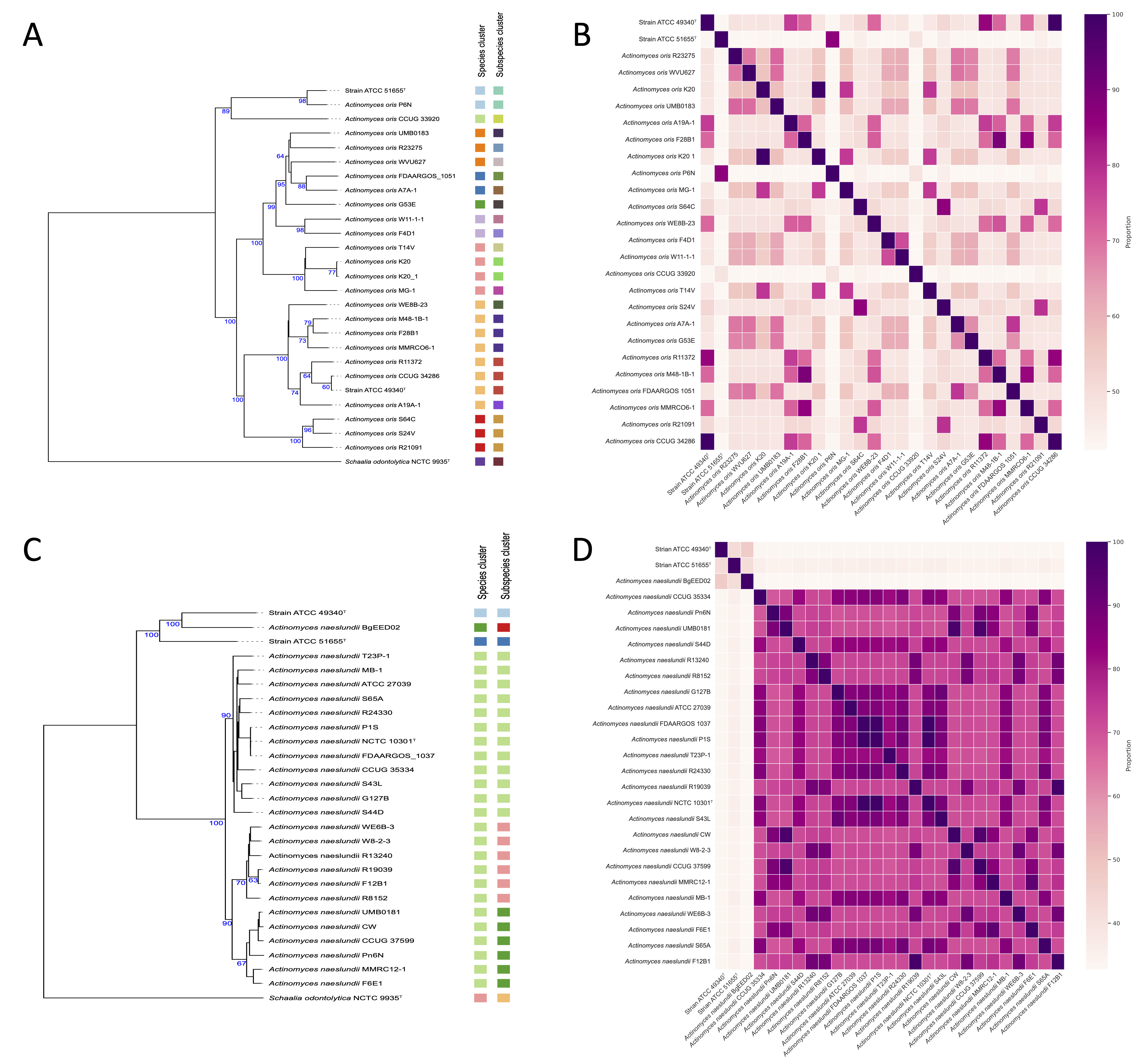
**

**Figure S2. Genome comparative analysis between two sequenced strains and available non-type strains of *Actinomyces oris* and *Actinomyces naeslundii*.** (A) Genome sequence-based tree based on TYGS analysis results for two sequenced strains and 24 *Actinomyces oris* non-type strains, where species cluster denotes groupings formed using a 70% dDDH threshold, and subspecies cluster indicates groupings established with a more stringent 79% dDDH threshold. *Schaalia odontolytica* NCTC 9935^T^ was employed as an outgroup. (B) Heatmap with dDDH value between two sequenced strains and 24 *Actinomyces* oris non-type strains, the dDDH values were calculated based on the confidence interval of formula *d_4_*. (C) Genome sequence-based tree based on TYGS analysis results for two sequenced strains and 24 *Actinomyces naeslundii* non-type strains, where species cluster denotes groupings formed using a 70% dDDH threshold, and subspecies cluster indicates groupings established with a more stringent 79% dDDH threshold. *Actinomyces naeslundii* NCTC 10301^T^ was a reference, and *Schaalia odontolytica* NCTC 9935^T^ was employed as an outgroup. (D) Heatmap with dDDH value between two sequenced strains and 24 *Actinomyces naeslundii* non-type strains, the dDDH values were calculated based on the confidence interval of formula *d_4_*. These genomes of non-type strains were sourced from the GenBank database in NCBI (Table S4).

**Table S1. The 16S rRNA gene sequences for the phylogenetic tree, nearly complete 16S rRNA gene sequence was downloaded from the NCBI database.**

| No | Strain | Accession number | Length (bp) |
| --- | --- | --- | --- |
| 1 | *Actinomyces naeslundii* Howell 279^T^ | ALJK01000050 | 1484 |
| 2 | *Actinomyces oris* CCUG 34288^T^ | AJ234053 | 1371 |
| 3 | *Actinomyces viscosus* NCTC 10951^T^ | X82453 | 1417 |
| 4 | *Actinomyces johnsonii* ATCC 49338^T^ | X81063 | 1417 |
| 5 | *Actinomyces bowdenii* M1327/96/1^T^ | AJ234039 | 1513 |
| 6 | *Actinomyces urogenitalis* DSM 15434^T^ | ACFH01000038 | 1461 |
| 7 | *Actinomyces weissii* 2298^T^ | FN552454 | 1379 |
| 8 | *Actinomyces marmotae* zg-325^T^ | MN173952 | 1503 |
| 9 | *Actinomyces howellii* NCTC 11636^T^ | X80411 | 1415 |
| 10 | *Actinomyces radicidentis* CCUG 36733^T^ | CP014228 | 1401 |
| 11 | *Actinomyces haliotis* WL80^T^ | KC196072 | 1459 |
| 12 | *Actinomyces slackii* CCUG 32792^T^ | AJ234067 | 1435 |
| 13 | *Actinomyces timonensis* DSM 23838^T^ | AKGF01000038 | 1464 |
| 14 | *Actinomyces bovis* NCTC 11535^T^ | X81061 | 1434 |
| 15 | *Actinomyces denticolens* DSM 20671^T^ | BDIO01000006 | 1469 |
| 16 | *Actinomyces oricola* CCUG 46090^T^ | AJ507295 | 1508 |
| 17 | *Actinomyces catuli* CCUG 41709^T^ | AJ276805 | 1400 |
| 18 | *Actinomyces gerencseriae* DSM 6844^T^ | AUBN01000027 | 1573 |
| 19 | *Actinomyces gaoshouyii* pika_113^T^ | MVIV01000003 | 1466 |
| 20 | *Actinomyces massiliensis* 4401292^T^ | AKIO01000026 | 1520 |
| 21 | *Actinomyces vulturis* VUL7^T^ | LZRK01000010 | 1456 |
| 22 | *Actinomyces israelii* CIP 103259^T^ | X82450 | 1441 |
| 23 | *Actinomyces graevenitzii* CCUG 27294^T^ | AJ540309 | 1413 |
| 24 | *Actinomyces procaprae* dk561^T^ | MH934916 | 1528 |
| 25 | *Actinomyces lilanjuaniae* 2129^T^ | CP032514 | 1549 |
| 26 | *Actinomyces wuliandei* 299^T^ | MN044992 | 1537 |
| 27 | *Actinomyces qiguomingii* 410^T^ | CP025228 | 1530 |
| 28 | *Actinomyces dentalis* R18165^T^ | AJ697609 | 1517 |
| 29 | *Actinomyces ruminicola* B71^T^ | DQ072005 | 1526 |
| 30 | *Actinomyces capricornis* MAS-1^T^ | LC570803 | 1533 |
| 31 | *Actinomyces trachealis* zg-993^T^ | MT894139 | 1518 |
| 32 | *Actinomyces respiraculi* ZJ750^T^ | MT614594 | 1521 |
| 33 | *Actinomyces faecalis* ZJ34^T^ | MT256397 | 1500 |
| 34 | *Schaalia odontolytica* CCUG 20536^T^ | NR_041983 | 1412 |
| 35 | Strain ATCC 49340^T^ | OQ981482 | 1300 |
| 36 | Strain ATCC 51655^T^ | OQ981481 | 1482 |

**Table S2. Six housekeeping gene information of all *Actinomyces* reference type strains used in housekeeping gene analysis and all housekeeping gene sequences were extracted from the results of RAST annotation. “+” presence, “-” absence.**

| **Strain name** | **Accession number** | ***atpA*** | ***rpoB*** | ***pgi*** | ***metG*** | ***gltA*** | ***gyrA*** |
| --- | --- | --- | --- | --- | --- | --- | --- |
| *Actinomyces naeslundii* NCTC 10301^T^ | GCF_001956585.1 | + | + | + | + | + | + |
| *Actinomyces oris* CCUG 34288^T^ | GCF_006546825.1 | + | + | + | + | + | + |
| *Actinomyces viscosus* NCTC 10951^T^ | GCF_900637975.1 | + | + | + | + | + | + |
| *Actinomyces johnsonii* CCUG 34287^T^ | GCF_006546835.1 | + | + | + | + | + | + |
| *Actinomyces bowdenii* DSM 15435^T^ | GCF_017592395.1 | + | + | + | + | + | + |
| *Actinomyces urogenitalis* DSM 15434^T^ | GCF_000159035.1 | + | + | + | + | + | + |
| *Actinomyces weissii* CCUG 61299^T^ | GCF_016598775.1 | + | + | + | + | + | + |
| *Actinomyces marmotae* zg-325^T^ | GCF_013177295.1 | + | + | + | + | + | + |
| *Actinomyces howellii* NCTC 11636^T^ | GCF_900637165.1 | + | + | + | + | + | + |
| *Actinomyces radicidentis* CCUG 36733^T^ | GCF_001553565.1 | + | + | + | + | + | + |
| *Actinomyces haliotis* JCM 18848^T^ | GCF_015355765.1 | + | + | + | + | + | + |
| *Actinomyces slackii* NCTC 11923^T^ | GCF_900637295.1 | + | + | + | + | + | + |
| *Actinomyces timonensis* DSM 23838^T^ | GCF_000295095.1 | + | + | + | + | + | + |
| *Actinomyces bovis* NCTC 11535^T^ | GCF_900444995.1 | + | + | + | + | + | + |
| *Actinomyces denticolens* DSM 20671^T^ | GCF_002072185.1 | + | + | + | + | + | + |
| *Actinomyces oricola* R5292^T^ | GCF_004104015.1 | + | + | + | + | + | + |
| *Actinomyces gerencseriae* DSM 6844^T^ | GCF_000429265.1 | + | + | + | + | + | + |
| *Actinomyces gaoshouyii* pika_113^T^ | GCF_002072175.1 | + | + | + | + | + | + |
| *Actinomyces massiliensis* 4401292^T^ | GCF_000269805.1 | + | + | + | + | + | + |
| *Actinomyces vulturis* VUL7^T^ | GCF_001687305.1 | + | + | + | + | - | + |
| *Actinomyces israelii* DSM 43320^T^ | GCF_000711965.1 | + | + | + | + | + | + |
| *Actinomyces procaprae* dk561^T^ | GCF_004798665.1 | + | + | + | + | + | + |
| *Actinomyces lilanjuaniae* 2129^T^ | GCF_003606385.1 | + | + | + | + | + | + |
| *Actinomyces wuliandei* 299^T^ | GCF_004010955.1 | + | + | + | + | + | + |
| *Actinomyces qiguomingii* 410^T^ | GCF_004102025.1 | + | + | + | + | + | + |
| *Actinomyces ruminicola* DSM 27982^T^ | GCF_900103885.1 | + | + | + | + | + | + |
| *Actinomyces dentalis* DSM 19115^T^ | GCF_000429225.1 | + | + | + | + | + | + |
| *Actinomyces trachealis* zg-993^T^ | GCF_015711475.1 | + | + | + | + | + | + |
| *Actinomyces respiraculi* ZJ750^T^ | GCF_014595995.2 | + | + | + | + | + | + |
| *Actinomyces faeculis* ZJ34^T^ | GCF_013184985.2 | + | + | + | + | + | + |
| *Schaalia odontolytica* CCUG 20536^T^ | GCF_900445025.1 | + | + | + | + | - | + |
| Strain ATCC 49340^T^ | JASPFC000000000 | + | + | + | + | + | + |
| Strain ATCC 51655^T^ | JASPEP000000000 | + | + | + | + | + | + |

***atpA:*** ATP synthase subunit alpha; ***rpoB:*** RNA polymerase, β-subunit; ***pgi:*** glucose-6-phosphate isomerase; ***metG:*** methionyl-tRNA synthetase; ***gltA:*** citrate synthase I; ***gyrA:*** DNA gyrase subunit A

**Table S3. Genome information of type strains used in genome comparative analysis, all genomes were performed a quality assessment using the gVolante2 with Busco v5.**

| **No** | **Strain Name** | **Genome accession numbers (RefSeq)** | **Genome Size (bp)** | **Completeness (%)** | **Contamination (%)** | **GC Content (%)** |
| --- | --- | --- | --- | --- | --- | --- |
| 1 | *Actinomyces naeslundii* NCTC 10301^T^ | GCF_001956585.1 | 3,119,690 | 100 | 0.47 | 67.94 |
| 2 | *Actinomyces oris* CCUG 34288^T^ | GCF_006546825.1 | 3,168,954 | 100 | 0.47 | 68.56 |
| 3 | *Actinomyces viscosus* NCTC 10951^T^ | GCF_900637975.1 | 3,491,241 | 100 | 1.07 | 68.91 |
| 4 | *Actinomyces johnsonii* CCUG 34287^T^ | GCF_006546835.1 | 3,348,294 | 100 | 0.95 | 67.52 |
| 5 | *Actinomyces bowdenii* DSM 15435^T^ | GCF_017592395.1 | 3,096,257 | 100 | 0.47 | 71.81 |
| 6 | *Actinomyces urogenitalis* DSM 15434^T^ | GCF_000159035.1 | 2,702,812 | 96.24 | 1.42 | 68.73 |
| 7 | *Actinomyces weissii* CCUG 61299^T^ | GCF_016598775.1 | 2,564,181 | 100 | 0.47 | 70.27 |
| 8 | *Actinomyces marmotae* zg-325^T^ | GCF_013177295.1 | 2,419,502 | 100 | 0.47 | 71.6 |
| 9 | *Actinomyces howellii* NCTC 11636^T^ | GCF_900637165.1 | 3,148,934 | 100 | 0.95 | 71.69 |
| 10 | *Actinomyces radicidentis* CCUG 36733^T^ | GCF_001553565.1 | 3,051,613 | 100 | 1.9 | 72.58 |
| 11 | *Actinomyces haliotis* JCM 18848^T^ | GCF_015355765.1 | 2,678,437 | 100 | 0.95 | 72.18 |
| 12 | *Actinomyces slackii* NCTC 11923^T^ | GCF_900637295.1 | 3,220,508 | 100 | 0.95 | 70.03 |
| 13 | *Actinomyces timonensis* DSM 23838^T^ | GCF_000295095.1 | 2,932,944 | 99.76 | 0.95 | 71.2 |
| 14 | *Actinomyces bovis* NCTC 11535^T^ | GCF_900444995.1 | 2,595,566 | 99.53 | 2.49 | 64.08 |
| 15 | *Actinomyces denticolens* DSM 20671^T^ | GCF_002072185.1 | 2,832,268 | 99.49 | 0 | 71.35 |
| 16 | *Actinomyces oricola* R5292^T^ | GCF_004104015.1 | 2,930,686 | 99.76 | 1.42 | 68.69 |
| 17 | *Actinomyces gerencseriae* DSM 6844^T^ | GCF_000429265.1 | 3,420,019 | 100 | 0.71 | 70.74 |
| 18 | *Actinomyces gaoshouyii* pika_113^T^ | GCF_002072175.1 | 2262740 | 100 | 0.47 | 71.15 |
| 19 | *Actinomyces massiliensis* 4401292^T^ | GCF_000269805.1 | 3,371,034 | 99.96 | 2.19 | 67.77 |
| 20 | *Actinomyces vulturis* VUL7^T^ | GCF_001687305.1 | 2,202,666 | 98.82 | 1.18 | 53.87 |
| 21 | *Actinomyces israelii* DSM 43320^T^ | GCF_000711965.1 | 4,025,772 | 100 | 1.78 | 71.44 |
| 22 | *Actinomyces procaprae* dk561^T^ | GCF_004798665.1 | 3,600,355 | 99.53 | 2.13 | 69.25 |
| 23 | *Actinomyces lilanjuaniae* 2129^T^ | GCF_003606385.1 | 3,025,012 | 96.76 | 0.47 | 69.02 |
| 24 | *Actinomyces wuliandei* 299^T^ | GCF_004010955.1 | 3,107,761 | 99.53 | 1.42 | 69.37 |
| 25 | *Actinomyces qiguomingii* 410^T^ | GCF_004102025.1 | 3,988,549 | 99.53 | 1.9 | 66.82 |
| 26 | *Actinomyces ruminicola* DSM 27982^T^ | GCF_900103885.1 | 3,103,556 | 99.53 | 0.95 | 69.91 |
| 27 | *Actinomyces dentalis* DSM 19115^T^ | GCF_000429225.1 | 3,531,393 | 99.53 | 2.25 | 73.11 |
| 28 | *Actinomyces trachealis* zg-993^T^ | GCF_015711475.1 | 2,526,250 | 99.53 | 0.71 | 65.77 |
| 29 | *Actinomyces respiraculi* ZJ750^T^ | GCF_014595995.2 | 2,912,106 | 99.76 | 2.01 | 70.12 |
| 30 | *Actinomyces faeculis* ZJ34^T^ | GCF_013184985.2 | 2701426 | 100 | 1.07 | 68.61 |
| 31 | *Schaalia odontolytica* NCTC 9935^T^ | GCF_900445025.1 | 2,454,426 | 99.76 | 0.95 | 64.86 |
| 32 | Strain ATCC 49340^T^ | JASPFC000000000 | 3,272,606 | 100% | 0.95 | 67.97 |
| 33 | Strain ATCC 51655^T^ | JASPEP000000000 | 3,084,459 | 100% | 0.47 | 68.07 |

**Table S4. Genome information of non-type strains of *Actinomyces oris* and *Actinomyces naeslundii* was used in genome comparative analysis; these genomes were sourced from the Genbank database in NCBI.**

| **No** | **Strain** | **Accession number** | **Genome Size (Mb)** | **Completeness (%)** | **Contamination (%)** | **GC Content (%)** |
| --- | --- | --- | --- | --- | --- | --- |
| 1 | *Actinomyces oris* K20 | GCA_000180155.1 | 2.9 | 89.34% | 1.32% | 68 |
| 2 | *Actinomyces oris* T14V | GCA_001553935.1 | 3 | 98.36% | 0% | 68 |
| 3 | *Actinomyces oris* MG-1 | GCA_001682715.1 | 2.9 | 95.72% | 1.77% | 68 |
| 4 | *Actinomyces oris* F4D1 | GCA_001929365.1 | 3 | 100% | 0% | 68.5 |
| 5 | *Actinomyces oris* S64C | GCA_001929375.1 | 3.1 | 99.53% | 0% | 68.5 |
| 6 | *Actinomyces oris* R21091 | GCA_001937365.1 | 3 | 100% | 0.08% | 68.5 |
| 7 | *Actinomyces oris* MMRCO6-1 | GCA_001937385.1 | 3.3 | 99.07% | 0% | 68 |
| 8 | *Actinomyces oris* M48-1B-1 | GCA_001937415.1 | 3.2 | 99.53% | 0% | 68 |
| 9 | *Actinomyces oris* F28B1 | GCA_001937425.1 | 3.3 | 100% | 0% | 68 |
| 10 | *Actinomyces oris* WE8B-23 | GCA_001937445.1 | 3.2 | 100% | 0% | 68 |
| 11 | *Actinomyces oris* CCUG 34286 | GCA_001937485.1 | 3.3 | 100% | 0% | 68 |
| 12 | *Actinomyces oris* R11372 | GCA_001937505.1 | 3.3 | 100% | 0% | 67.5 |
| 13 | *Actinomyces oris* R23275 | GCA_001937535.1 | 3.1 | 97.20% | 0% | 68 |
| 14 | *Actinomyces oris* W11-1-1 | GCA_001937545.1 | 3 | 99.25% | 0% | 68.5 |
| 15 | *Actinomyces oris* G53E | GCA_001937555.1 | 3 | 99.53% | 0.12% | 68.5 |
| 16 | *Actinomyces oris* S24V | GCA_001937655.1 | 3 | Inconclusive | Inconclusive | 68 |
| 17 | *Actinomyces oris* P6N | GCA_001937665.1 | 3.1 | 99.07% | 0% | 68 |
| 18 | *Actinomyces oris* CCUG 33920 | GCA_001937675.1 | 3 | 100% | 0% | 68 |
| 19 | *Actinomyces oris* A7A-1 | GCA_001937715.1 | 3.1 | 100% | 0% | 69 |
| 20 | *Actinomyces oris* A19A-1 | GCA_001937725.1 | 3.3 | 99.53% | 0% | 68 |
| 21 | *Actinomyces oris* UMB0183 | GCA_002847555.1 | 3 | 100% | 0% | 68.5 |
| 22 | *Actinomyces oris* FDAARGOS_1051 | GCA_016127955.1 | 3.2 | 100% | 0% | 68 |
| 23 | *Actinomyces oris* K20-1 | GCA_023169925.1 | 3.1 | 100% | 0% | 68 |
| 24 | *Actinomyces oris* WVU627 | GCA_027945475.1 | 3.1 | 99.81% | 0% | 68.5 |
| 25 | *Actinomyces naeslundii* R19039 | GCA_001937475.1 | 3.2 | 95.26% | 2.48% | 67.5 |
| 26 | *Actinomyces naeslundii* W8-2-3 | GCA_001937595.1 | 3.2 | 95.46% | 3.07% | 67.5 |
| 27 | *Actinomyces naeslundii* R13240 | GCA_001937605.1 | 3.2 | 95.24% | 2.90% | 68 |
| 28 | *Actinomyces naeslundii* R8152 | GCA_001937615.1 | 3.2 | 95.29% | 3% | 67.5 |
| 29 | *Actinomyces naeslundii* WE6B-3 | GCA_001937735.1 | 3.2 | 94.78% | 2.88% | 67.5 |
| 30 | *Actinomyces naeslundii* F12B1 | GCA_001956355.1 | 3.2 | 95.26% | 2.39% | 68 |
| 31 | *Actinomyces naeslundii* Pn6N | GCA_001956365.1 | 3.2 | 94.22% | 1.50% | 67.5 |
| 32 | *Actinomyces naeslundii* MMRC12-1 | GCA_001956415.1 | 3.2 | 94.14% | 1.65% | 67.5 |
| 33 | *Actinomyces naeslundii* F6E1 | GCA_001956435.1 | 3 | 85.98% | 1.49% | 67.5 |
| 34 | *Actinomyces naeslundii* CCUG 37599 | GCA_001956445.1 | 3.2 | 94.01% | 1.57% | 68 |
| 35 | *Actinomyces naeslundii* T23P-1 | GCA_001956475.1 | 3.1 | 94.46% | 3.88% | 68 |
| 36 | *Actinomyces naeslundii* S44D | GCA_001956485.1 | 3.1 | 95.74% | 2.17% | 67.5 |
| 37 | *Actinomyces naeslundii* R24330 | GCA_001956505.1 | 3.1 | 95.89% | 1.52% | 67.5 |
| 38 | *Actinomyces naeslundii* S43L | GCA_001956515.1 | 3.1 | 96.34% | 1.43% | 67.5 |
| 39 | *Actinomyces naeslundii* MB-1 | GCA_001956555.1 | 3.1 | 93.59% | 1.73% | 68 |
| 40 | *Actinomyces naeslundii* CCUG 35334 | GCA_001956565.1 | 3.2 | 96.42% | 0.37% | 67.5 |
| 41 | *Actinomyces naeslundii* S65A | GCA_001956575.1 | 3.2 | 96.03% | 2.78% | 67.5 |
| 42 | *Actinomyces naeslundii* G127B | GCA_001956635.1 | 3.1 | 97.02% | 1.39% | 68 |
| 43 | *Actinomyces naeslundii* UMB0181 | GCA_002847585.1 | 3.1 | 94.20% | 0.78% | 67.5 |
| 44 | *Actinomyces naeslundii* FDAARGOS_1037 | GCA_016127855.1 | 3.2 | 98.18% | 1.94% | 67.5 |
| 45 | *Actinomyces naeslundii* ATCC 27039 | GCA_023170065.1 | 3.2 | 97.19% | 2.66% | 67.5 |
| 46 | *Actinomyces naeslundii* CW | GCA_026802095.1 | 3.1 | 94.42% | 0.96% | 67.5 |
| 47 | *Actinomyces naeslundii* P1S | GCA_031296715.1 | 3.1 | 97.06% | 1.88% | 67.5 |
| 48 | *Actinomyces naeslundii* BgEED02 | GCA_901873715.1 | 3.1 | 97.99% | 2.46% | 68 |

**Table S5. The genomic islands (GIs) distribution statistic of three *Actinomyces* strains**

| **Strain** | **GIs** | **Island start** | **Island end** | **Length (bp)** | **Gene number** |
| --- | --- | --- | --- | --- | --- |
| Strain ATCC 49340^T^ | GI1 | 272,084 | 279,197 | 7,113 | 7 |
|  | GI2 | 596,468 | 658,763 | 62,295 | 67 |
|  | GI3 | 651,448 | 655,559 | 4,111 | 8 |
|  | GI4 | 678,325 | 682,988 | 4,663 | 4 |
|  | GI5 | 845,415 | 850,769 | 5,354 | 8 |
|  | GI6 | 927,705 | 933,886 | 6,181 | 9 |
|  | GI7 | 1,073,166 | 1,090,300 | 17,134 | 12 |
|  | GI8 | 1,100,805 | 1,129,896 | 29,091 | 29 |
|  | GI9 | 1,103,516 | 1,118,341 | 14,825 | 15 |
|  | GI10 | 1,244,439 | 1,251,099 | 6,660 | 4 |
|  | GI11 | 1,379,791 | 1,385,480 | 5,689 | 7 |
|  | GI12 | 1,419,143 | 1,428,946 | 9,803 | 5 |
|  | GI13 | 1,432,702 | 1,437,417 | 4,715 | 8 |
|  | GI14 | 1,709,099 | 1,731,453 | 22,354 | 18 |
|  | GI15 | 1,717,628 | 1,727,853 | 10,225 | 8 |
|  | GI16 | 2,420,966 | 2,425,965 | 4,999 | 3 |
|  | GI17 | 2,606,413 | 2,622,881 | 16,468 | 19 |
|  | GI18 | 2,736,606 | 2,769,168 | 32,562 | 29 |
|  | GI19 | 2,745,462 | 2,749,999 | 4,537 | 5 |
|  | GI20 | 3,129,004 | 3,134,491 | 5,487 | 2 |
|  | GI21 | 3,305,498 | 3,373,050 | 67,552 | 48 |
|  | GI22 | 3,336,071 | 3,342,281 | 6,210 | 6 |
|  | GI23 | 3,344,951 | 3,350,914 | 5,963 | 4 |
|  | GI24 | 3,360,845 | 3,365,060 | 4,215 | 3 |
| Strain ATCC 51655^T^ | GI1 | 256,376 | 261,047 | 4,671 | 5 |
|  | GI2 | 438,953 | 463,289 | 24,336 | 20 |
|  | GI3 | 440,934 | 460,571 | 19,637 | 10 |
|  | GI4 | 634,608 | 656,176 | 21,568 | 24 |
|  | GI5 | 1,163,515 | 1,181,321 | 17,806 | 13 |
|  | GI6 | 1,371,281 | 1,378,314 | 7,033 | 5 |
|  | GI7 | 1,524,503 | 1,546,123 | 21,620 | 22 |
|  | GI8 | 1,535,796 | 1,543,277 | 7,481 | 8 |
|  | GI9 | 1,664,011 | 1,690,995 | 26,984 | 24 |
|  | GI10 | 1,667,172 | 1,674,872 | 7,700 | 5 |
|  | GI11 | 1,683,570 | 1,690,995 | 7,425 | 7 |
|  | GI12 | 1,760,982 | 1,766,856 | 5,874 | 7 |
|  | GI13 | 1,895,950 | 1,904,375 | 8,425 | 10 |
|  | GI14 | 2,746,766 | 2,757,196 | 10,430 | 11 |
|  | GI15 | 3,128,292 | 3,139,940 | 11,648 | 11 |
|  | GI16 | 3,133,039 | 3,137,702 | 4,663 | 8 |
| *Actinomyces oris* CCUG 34288^T^ | GI1 | 244,334 | 250,082 | 5,748 | 9 |
|  | GI2 | 482,058 | 501,695 | 19,637 | 24 |
|  | GI3 | 542,964 | 548,791 | 5,827 | 7 |
|  | GI4 | 818,843 | 825,942 | 7,099 | 10 |
|  | GI5 | 963,734 | 968,068 | 4,334 | 5 |
|  | GI6 | 1,037,754 | 1,044,818 | 7,064 | 5 |
|  | GI7 | 1,126,250 | 1,129,533 | 3,283 | 7 |
|  | GI8 | 1,205,780 | 1,215,488 | 9,708 | 4 |
|  | GI9 | 1,227,607 | 1,234,935 | 7,328 | 6 |
|  | GI10 | 1,354,807 | 1,360,603 | 5,796 | 6 |
|  | GI11 | 1,374,221 | 1,382,345 | 8,124 | 13 |
|  | GI12 | 1,374,221 | 1,382,004 | 7,783 | 12 |
|  | GI13 | 1,530,814 | 1,534,947 | 4,133 | 9 |
|  | GI14 | 1,546,844 | 1,554,871 | 8,027 | 14 |
|  | GI15 | 1,582,582 | 1,593,479 | 10,897 | 10 |
|  | GI16 | 1,636,884 | 1,646,889 | 10,005 | 12 |
|  | GI17 | 2,457,187 | 2,519,620 | 62,433 | 64 |
|  | GI18 | 2,464,354 | 2,468,909 | 4,555 | 7 |
|  | GI19 | 2,478,309 | 2,483,916 | 5,607 | 6 |
|  | GI20 | 2,486,609 | 2,490,666 | 4,057 | 5 |
|  | GI21 | 2,495,193 | 2,514,152 | 18,959 | 20 |

**Table S6. Pathogenic analysis results using the PathogenFinder online tool.**

| **Strain** | **Strain ATCC 49340^T^** | **Strain ATCC 51655^T^** | ***Actinomyces oris* CCUG 34288^T^** |
| --- | --- | --- | --- |
| **Human pathogen** | Yes | Yes | Yes |
| **Matched Family** | 1 | 1 | 1 |
| **Organisms** | *Cutibacterium acnes* (formerly *Propionibacterium acnes*) KPA171202 | *Cutibacterium acnes* (formerly *Propionibacterium acnes*) KPA171202 | *Cutibacterium acnes* (formerly *Propionibacterium acnes*) KPA171202 |
| **Class** | *Actinomycetes* | *Actinomycetes* | *Actinomycetes* |
| **Protein function** | amidinotransferase | amidinotransferase | amidinotransferase |
| **Identity (%)** | 88.53 | 88.8 | 88.8 |

**Table S7. CRISPRs distribution of three analyzed *Actinomyces* strains.**

| **Strain** | **Element** | **CRISPR Id** | **Start** | **End** | **Spacer count** |
| --- | --- | --- | --- | --- | --- |
| Strain ATCC 49340^T^ | CRISPR | 1A7_00008_1 | 11376 | 11526 | 2 |
|  | CRISPR | 1A7_00008_2 | 12552 | 17923 | 87 |
|  | CRISPR | 1A7_00010_1 | 63225 | 63377 | 2 |
|  | CRISPR | 1A7_00010_2 | 63468 | 64107 | 10 |
|  | CRISPR | 1A7_00019_1 | 55699 | 55789 | 1 |
|  | CRISPR | [1A7_00022_1](https://crisprcas.i2bc.paris-saclay.fr/CrisprCasFinder/Viewing/638242615048292569) | 3343 | 3817 | 6 |
|  | CRISPR | [1A7_00022_2](https://crisprcas.i2bc.paris-saclay.fr/CrisprCasFinder/Viewing/638242615048292569) | 5914 | 6381 | 6 |
|  | CRISPR | [1A7_00022_3](https://crisprcas.i2bc.paris-saclay.fr/CrisprCasFinder/Viewing/638242615048292569) | 6712 | 7187 | 6 |
|  | CRISPR | [1A7_00022_4](https://crisprcas.i2bc.paris-saclay.fr/CrisprCasFinder/Viewing/638242615048292569) | 21154 | 21479 | 4 |
|  | CRISPR | [1A7_00022_5](https://crisprcas.i2bc.paris-saclay.fr/CrisprCasFinder/Viewing/638242615048292569) | 21848 | 23266 | 19 |
|  | CRISPR | [1A7_00034_1](https://crisprcas.i2bc.paris-saclay.fr/CrisprCasFinder/Viewing/638242615048292569) | 19726 | 19814 | 1 |
|  | CRISPR | [1A7_00035_1](https://crisprcas.i2bc.paris-saclay.fr/CrisprCasFinder/Viewing/638242615048292569) | 21352 | 21441 | 1 |
|  | CRISPR | [1A7_00037_1](https://crisprcas.i2bc.paris-saclay.fr/CrisprCasFinder/Viewing/638242615048292569) | 20998 | 21668 | 9 |
|  | CRISPR | [1A7_00056_1](https://crisprcas.i2bc.paris-saclay.fr/CrisprCasFinder/Viewing/638242615048292569) | 501 | 589 | 1 |
| Strain ATCC 51655^T^ | CRISPR | 1A31_00001_crispr_1 | 236421 | 236530 | 1 |
|  | CRISPR | 1A31_00020_1 | 42631 | 42750 | 1 |
|  | CRISPR | 1A31_00022_crispr_1 | 6321 | 6484 | 2 |
| *Actinomyces oris* CCUG 34288^T^ | CRISPR | NZ_VICC01000001_1 | 386715 | 387536 | 13 |
|  | CRISPR | NZ_VICC01000004_1 | 307737 | 308000 | 5 |
|  | CRISPR | NZ_VICC01000006.1_crispr_1 | 231194 | 231303 | 1 |

**Table S8. Cas gene distribution of three analyzed *Actinomyces* strains**

| **Strain** | **Element** | **Cas Type** | **Start** | **End** | **Gene** | **Cas genes** |
| --- | --- | --- | --- | --- | --- | --- |
| Strain ATCC 49340^T^ | Cas cluster | General-Class1 | 18,366 | 27,170 | 7 | Cas1_0_IE, Cas3_0_I, Cas5_0_IE, Cas6_0_IE, Cas7_0_IE, Cse1_0_IE, Cse2_0_IE |
|  | Cas cluster | General-Class1 | 56,343 | 63,209 | 3 | Cas3_0_IU, Csb1_0_IU, Csb2_0_IU |
|  | Cas cluster | [General-Class1](https://crisprcas.i2bc.paris-saclay.fr/CrisprCasFinder/Viewing/638242615048292569) | 10,952 | 24,536 | 5 | Cas10_0_III, Cas2_0_I-II-III, Cas6_0_I-III, Csm3_1_IIIAD, Csm3_1_IIIAD |
|  | Cas cluster | [General-Class1](https://crisprcas.i2bc.paris-saclay.fr/CrisprCasFinder/Viewing/638242615048292569) | 21,848 | 22,147 | 1 | Cas2_0_I-II-III-V |
|  | Cas cluster | [General-Class2](https://crisprcas.i2bc.paris-saclay.fr/CrisprCasFinder/Viewing/638242615048292569) | 22,147 | 23,754 | 1 | Cas1_0_I-II-III-V |
| Strain ATCC 51655^T^ | Cas cluster | General-Class1 | 47,971 | 48,756 | 1 | Cas4_0_I-II |
|  | Cas cluster | General-Class1 | 43,758 | 45,899 | 1 | Cas3_0_I |
| *Actinomyces oris* CCUG 34288^T^ | Cas cluster | General-Class1 | 379,818 | 386,696 | 3 | Cas3_TypeIU, Csb1_TypeIU, Csb2_TypeIU |
|  | Cas cluster | General-Class1 | 70,192 | 73,941 | 1 | Cas3_0_I |
|  | Cas cluster | General-Class1 | 421,203 | 599,477 | 2 | Cas2_0_I-II-III-V, Cas4_0_I-II |
